# Stress in native grasses under ecologically relevant heat waves

**DOI:** 10.1371/journal.pone.0204906

**Published:** 2018-10-11

**Authors:** Michael Davies, Heath Ecroyd, Sharon A. Robinson, Kristine French

**Affiliations:** 1 Centre for Sustainable Ecosystem Services, University of Wollongong, Wollongong, NSW, Australia; 2 School of Biological Sciences, University of Wollongong, Wollongong, NSW, Australia; 3 Illawarra Health and Medical Research Institute, Wollongong, NSW, Australia; USDA Agricultural Research Service, UNITED STATES

## Abstract

Future increases in the intensity of heat waves (high heat and low water availability) are predicted to be one of the most significant impacts on organisms. Using six native grasses from Eastern Australia, we assessed their capacity to tolerate heat waves with low water availability. We were interested in understanding differential response between native grasses of differing photosynthetic pathways in terms of physiological and some molecular parameters to ecologically relevant summer heat waves that are associated with low rainfall. We used a simulation heatwave event in controlled temperature cabinets and investigated effects of the different treatments on four stress indicators: leaf senescence, leaf water content, photosynthetic efficiency and the relative expression of two heat shock proteins, Hsp70 and smHsp17.6. Leaf senescence was significantly greater under the combined stress treatment, while declines in leaf water content and photosynthetic efficiency were much larger for C3 than C4 plants, particularly under the combined stress treatment. Species showed an increase in expression of Hsp70 associated with heat treatment, rather than drought stress. In contrast Hsp17.6 was only detected in two species, responding to heat rather than drought, although species’ responses were variable. Overall, the C3 species were less tolerant than C4 species. Variation in individual plants within species was evident, especially under multiple stresses, and indicates that losses of individual plants may occur during a heat wave associated with this variability in tolerance. Heat waves will impose significant stress on plant communities that would not otherwise occur when heat and drought stress are experienced singly. Using ecologically relevant heat stress is likely to yield better predictability of how native plants will cope under a hotter, drier future.

## Introduction

Global temperatures are estimated to rise by 1–4.5°C over the next 100 years under the various models of carbon emissions [1, 2]. One of the major outcomes of a large-scale climatic shift will be an increase in extreme weather events such as heat waves, droughts, floods, cyclones and wildfires [2]. Increases in the intensity, duration and frequency of both heat waves and drought events are expected to occur globally and, in some areas, this has already taken place [2, 3].

Heat waves often occur during periods of significant water shortages where there is a build-up of temperature associated with little rainfall, and given the climatic predictions, it is reasonable to assume that the frequency with which these two stresses coincide will increase. Heat waves associated with dry conditions cause significantly greater stress than either individual stressor alone [4]. Studies on agricultural crops report substantial declines in productivity after exposure to combined heat stress and drought [4–9]. Few studies have examined the influence that varying temperatures and water availability may impose on native plant species, however, De Boeck *et al*. (2010) has shown that heat waves combined with drought events impose a decrease in growth and functionality on some herb communities [10].

Stress can be measured using a range of characteristics that include ecological, physiological and molecular parameters. We have chosen four measures of stress that include physiological and molecular measures that are indicative of impacts immediately following a single ecologically relevant heat wave. When plants are drought stressed, the proportion of water in leaves is lowered and assessment of leaf water content (LWC) is one of the most direct measurements of the extent to which drought stress is affecting a plant and is therefore an excellent indicator of plant health [11]. Increasing climatic extremes such as heatwaves result in decreased biomass [12–14] which may be due to lower photosynthate accumulation through lowering of photosynthetic efficiency, leaf senescence, or both. Leaf senescence is commonly seen in plants under stress (e.g. [15]) and is a common response in grasses. Because the occurrence of leaf senescence may be due to either an adaptive strategy to produce more heat-tolerant leaves or an inability to tolerate environmental conditions, it cannot be used to infer how a plant is tolerating the environmental stress directly [16], however, such responses suggest that heat waves are affecting plant allocation choices.

The constituents of the photosynthetic apparatus are highly sensitive to stress, and disruption of these photosystems can lead to impaired growth, function and reproduction in plants [17]. PSII efficiency (measured as F_v_/F_m_) is directly proportional to the quantum efficiency of oxygen evolution by PSII and thus is a direct indicator of photosynthetic efficiency [18]. Stress tolerance can therefore be inferred from this measurement as a higher (or lower) F_v_/F_m_ value will be indicative of a greater (or lesser) tolerance respectively. Since chlorophyll fluorescence can be measured quickly it is thus a practical way to compare heat damage in multiple species and with appropriate data replication [19, 20].

Finally, one of the most significant effects of heat stress at a molecular level is protein misfolding and aggregation in cells [4, 21, 22]. A key adaptation utilised by all organisms to tolerate this is the up-regulation of heat shock proteins (Hsps) which facilitate refolding of proteins in cells. Their role has been reviewed in detail [22–26]. While often constitutively expressed under basal conditions in plant cells, Hsp abundance increases dramatically (up to 200 fold for low molecular weight Hsps) as a protective mechanism during cellular stress [26]. A direct positive correlation exists between increased Hsp expression and increased thermotolerance [24], however the application of heat stress in plant Hsp studies has often been over short acute time periods [27, 28]. These acute stress experiments bear little ecological relevance to how Hsps are utilised under naturally occurring environmental stress. In order to accurately represent stress of ecological relevance, experimental studies on heat waves should closely follow regional climatic characteristics [10, 29] and it is logical to assume that naturally-occurring species that have evolved in warmer environments (e.g. C4 and CAM plant species) will show superior utilisation of Hsps as a mechanism to tolerate heat stress.

We performed a simulation heatwave treatment and measured 4 response variables of stress in plants to investigate the effects of heat and drought stress on native grasses. Grasses of both C3 and C4 photosynthetic pathways were used to compare the response of species with differing levels of tolerance to such climatic conditions. The response variables measured were LWC, leaf senescence, photosynthetic efficiency (F_v_/F_m_) and the expression of two heat shock proteins (Hsp70 & smHsp17.6). Whilst Hsp70 is a key component of the refolding machinery in cells [30], smHsp17.6 is likely to be one of the initial proteins that binds to misfolded proteins, in order to stabilise them and facilitate their subsequent refolding [31].

## Materials and methods

### Study species & growth conditions

Six native grass species were selected; three C3 and three C4 species, from a range of different tribes from the Poaceae family as stress responses are known to vary with phylogeny [32]. The three C3 species were *Poa labillardierei* (tribe Poaeae; hereafter *Poa*), *Austrostipa ramosissima* (tribe Stipeae; hereafter *Austrostipa*) and *Microlaena stipoides* (tribe Ehrharteae; hereafter *Microlaena*), and the three C4 species were *Themeda triandra* (tribe Andropogoneae; hereafter *Themeda*), *Imperata cylindrica* (tribe Andropogoneae; hereafter *Imperata*) and *Eragrostis elongata* (tribe Eragrostideae; hereafter *Eragrostis*). All plants were sourced from Green Plan Nursery and Jamberoo Native Nursery and potted into round 15cm pots with the same 1:1 ratio of sand: top-soil potting mix (RICHGRO, Perth). Plants were kept at the Ecological Research Facility, University of Wollongong (34.40°S, 150.88°E) between April and August 2015 and were watered to field capacity every day prior to experimentation. Plants were of unknown age as they were bought to be of similar size, but plants were likely to be just prior to their first flowering season. Sale-ready plants in these sized pots tend to be on the verge of maturity when sold.

### Design of stress treatments

In June 2015, twenty-four replicates of each species were placed into growth cabinets (Model TPG-2400-TH-CO2, Thermoline, Perth, Western Australia) and acclimated at control conditions (outlined below) for one week prior to experimental treatments. Growth cabinets were used to manipulate the ambient temperatures experienced by the plants, while manipulation of water availability was performed manually. In order to measure the total proportion of leaf senescence that occurred during the experimental treatments, all dead material was pruned prior to the experiment starting, leaving only living plant material.

All growth cabinets had a 12-h photoperiod (06:00–18:00) with a maximum illumination of 850 μmols.m^-2^.s^-1^ and 2 h of ramping at either end of the day simulating dawn (06:00–08:00) and dusk (16:00–18:00). Temperature ramp rates (°C/h) were set so that 4 h of ramping occurred from night to day (06:00–10:00) and day to night (18:00–22:00). This provided 8 h of maximum (day time) and minimum (night time) temperatures daily. Relative humidity was set at 60% for all treatments, representing the annual mean relative humidity according to the weather station located at the University of Wollongong (Bureau of Meteorology). All treatments were carried out simultaneously over an eleven-day period on all six species, with six biological replicates per treatment.

#### Control (C)

Day and night-time temperatures were set to 25°C and 18°C respectively (ramp rate of 1.75°C/h). Control plants received 200 mL of water every day, ensuring all pots reached field capacity, and that these plants were not limited by water availability.

#### Drought stress (D)

The drought stress treatment was modelled on previous studies [6–8, 33] and a pilot study to ensure LWC was lowered. Starting on Day 1, drought-treated plants received no water for the duration of the experiment (11 days). Day and night-time temperatures were the same as in the control treatment.

#### Heat stress (H)

The heat stress treatment was modelled to represent an Australian heat wave located within the temporal coastal climatological context of southern New South Wales, Australia. The simulation heatwave was modelled on the Bureau of Meteorology’s definition of a heatwave as three or more days of unusually high maximum and minimum temperatures. Plants experienced a single four day heat wave event commencing on Day 8 and ending on Day 11. The heat stress commenced with day and night-time temperatures of 32°C and 25°C respectively (Day 8). This was followed by three consecutive days and nights at 39°C and 25°C respectively (Days 8–11). Temperature ramp rates were set at 1.75°C/h for the first night & day and 3.5°C/h for the three subsequent days. Heat stress plants received equal water volumes to control plants for the duration of the 11 days.

#### Heat + Drought stress (HD)

This treatment was performed by subjecting a group of drought-treated plants to the heat stress treatment. The drought stress was applied for the full 11 days and the heat stress was applied on days 8–11, as described above. This enabled a combined heat and drought stress, simulating a heat wave under drought conditions.

### Physiological and morphological stress measurements

Plant chlorophyll fluorescence (F_v_/F_m_) was used to measure photosynthetic efficiency (PAM2100; Heinz-Walz, Effeltrich, Germany). Measurements were taken on Day 0 prior to the stress treatments and on Day 11, directly after the completion of treatments. Plants were given 1 h of dark adaptation prior to all measurements. Three separate measurements were taken on the healthiest leaves and the average of these measurements was designated as the plant’s overall fluorescence reading.

To compare the proportional leaf senescence amongst treatments, living and dead material for each plant was separated at the end of the experiment, oven dried (Contherm Series Five Oven, Petone) at 60°C for five days and reweighed. A ratio of dead biomass relative to total biomass (living plus dead) was calculated. For LWC, living plant material was weighed separately at the time of sampling (fresh weight) and directly after being oven-dried (dry weight). From this we calculated (FW-DW)/FW, where FW = fresh weight, and DW = subsequent dry weight of the plant material.

### Measurements of Hsp70 and Hsp17.6 expression

Leaf samples for analysis of Hsp expression were taken directly after fluorescence measurements. Approximately 500 mg of healthy living leaf material per plant was weighed and then snap frozen in liquid nitrogen and stored at -80°C. Protein extraction was performed using a modified method of the SDS-buffer extraction protocol from Knight and Ackerly (2001) [34]. Briefly, soluble proteins were extracted from the leaf samples using SDS-buffer (100 mM Tris, 2.5% w/v SDS, 5 mM EDTA, 0.2 mM PMSF, pH 8.0) with 1 mL of SDS-extraction buffer added per 100 mg of freeze-dried plant material prior tissue lysing (TissueLyser II, Qiagen, Melbourne, Australia). Samples were boiled at 95°C for 5 min and left to stand at room temperature for 1 h before centrifuging (20,000 x *g*, 10 min, 4°C). Following centrifugation, the supernatant was retained, mixed with SDS-PAGE loading buffer (final concentrations 50 mM Tris-HCl, 2% w/v SDS, 10% v/v glycerol, 0.1% w/v bromophenol blue and 2% v/v β-mercaptoethanol, pH 6.8) and 10 μL of each protein sample subjected to SDS-PAGE using Mini-PROTEAN TGX Stain-Free Gels (Bio-Rad, Hercules) for 1 h at 120V. A sample of leaf material from an individual *E*. *elongata* previously found to express both Hsp70 and Hsp17.6, was used as a positive control and molecular weight markers (Precision plus protein dual standards; Bio-Rad, Hercules) were also loaded onto all gels. Gels were imaged using a Bio-Rad ChemiDocMP imager (Bio-Rad, Hercules) in order to quantify the relative protein content in each lane of the gel.

Samples were then transferred from gels to polyvinylidene difluoride (PVDF) membranes (Bio-Rad, Hercules) at 100 V for 1 h using standard cold wet blotting techniques [35]. Membranes were blocked with 5% (w/v) skim milk in Tris-buffered saline (TBS; 10 mM Tris HCL, 150 mM NaCl, pH 7.5) for 1 h at room temperature (23°C). Membranes were cut in half at the 37 kDa marker, rinsed and incubated with primary antibodies diluted 1:2000 with 5% (w/v) skim milk in TBS-T (TBS containing 0.1% Tween-20) over night at 4°C. Primary antibodies used were mouse anti-Hsp70 monoclonal antibody (N27F3-4; ENZO Life Sciences Inc., Farmingdale) and rabbit anti-Hsp17.6 polyclonal antibody (ab80183) (Abcam, Cambridge, UK). Membranes were washed (3 x 10 min washes in TBS-T) before being incubated with peroxidase conjugated secondary antibodies. Secondary antibodies (rabbit anti-mouse for Hsp70 or goat anti-rabbit for Hsp17.6) (Sigma-Aldrich Co., St. Louis, USA) were diluted 1:2000 in TBS-T containing 5% (w/v) skim milk and incubated for 1 h at room temperature. Antibodies were previously found to detect a band of the correct molecular mass (~70 kDa for the anti-Hsp70 antibody and ~17 kDa for the anti-Hsp17.6 antibody) in leaf samples from each species used in this work that had been subjected to an acute heat stress of 45°C for 6 h in a drying oven (Contherm Series Five Oven, Petone).

Protein bands were detected using western ECL blotting detection reagents (GE Healthcare, Parramatta, N.S.W, Australia) and exposure of the membrane to film (Amersham Hyperfilm, GE Healthcare, Parramatta, N.S.W, Australia) according to the manufacturer’s instructions. The film was imaged using a Gel Logic 2200 Pro system with Carestream Molecular Imaging Software 5.0 (Carestream, Rochester), and analysis was performed via densitometry on the resulting images using Image Studio Lite (LI-COR, Lincoln).

**Fig 1:**
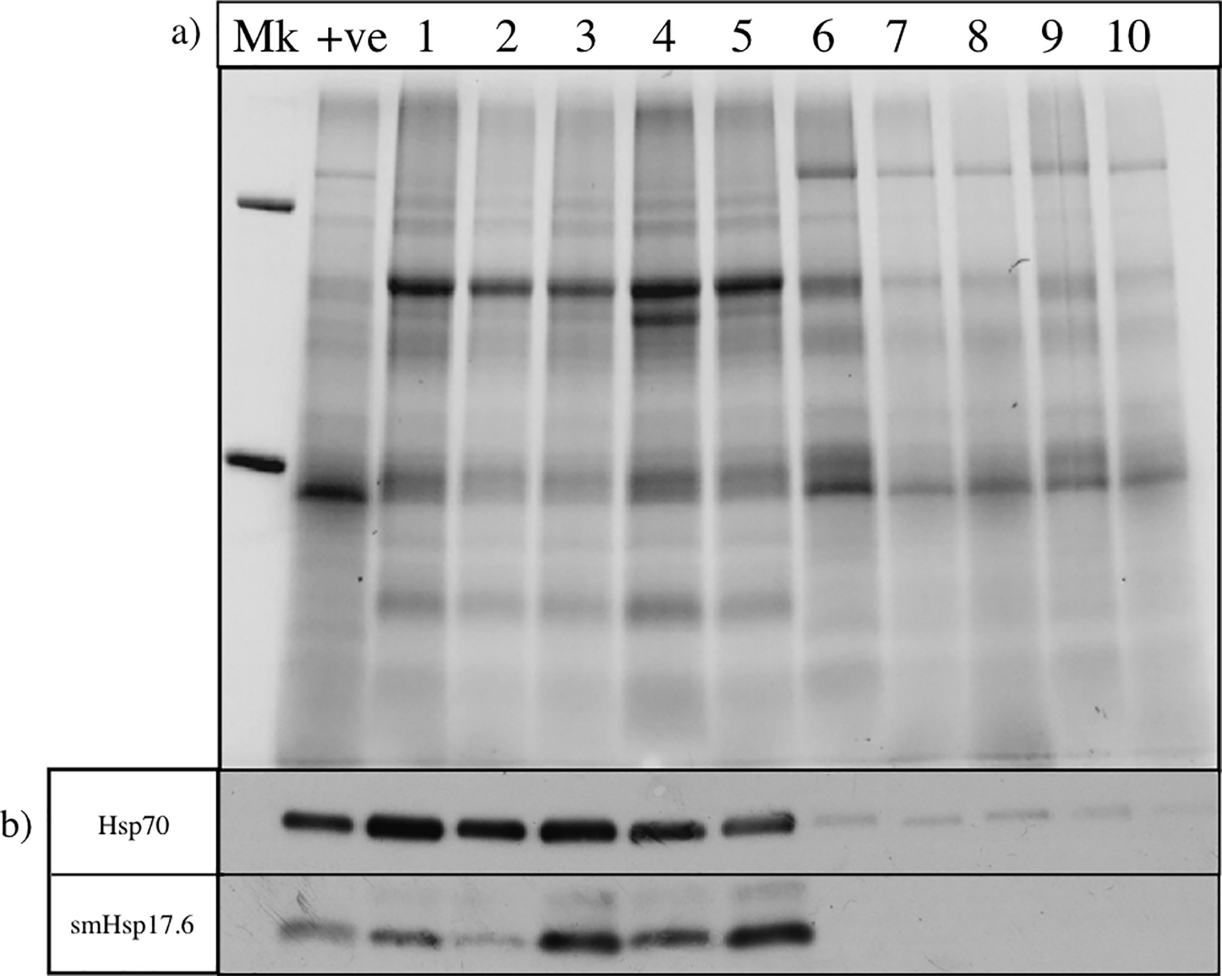
Leaf protein extracts from *Microlaena stipoides* and *Eragrostis elongata* after a heat stress treatment. Lane ‘Mk’ = Weight marker (kDa), lane ‘+ve’ = positive control used to calculate relative protein expression within the immunoblots as well as allow for standardised comparison across immunoblots. Lanes 1–5 = biological replicates of protein extract samples of *Microlaena stipoides* after a heat stress treatment. Lanes 6–10 = biological replicates of protein extract samples from *Eragrostis elongata* after a heat stress treatment. a) SDS-PAGE gel, b) Immunoblot for Hsp70 and smHsp 17.6 corresponding to the above SDS-PAGE gel.

We used the relative amount of protein (normalized to a control sample that was run on every gel) to compare expression levels of the Hsps between species and treatments. We used this approach rather than the expression of a housekeeping protein, such as GADPH, as these levels likely vary between species and with treatment. We therefore considered total protein content as a more reliable measure of relative loading as it does not depend on the expression of a single protein [36,37].Densitometry values were normalised between immunoblots by dividing the intensity of the protein signal (density) in each sample by the density of the positive control (*E*. *elongata*). To standardise protein expression by the amount of protein loaded, each density value was then normalised by the relative loading of its corresponding lane from the previously imaged gel (Fig 1). The equation used to calculate relative expression of Hsps was therefore: (Si/Ci) / (Sg/Cg) = Relative protein expression,

where:

Sg = Density of total protein in sample lane from SDS-PAGE gel

Cg = Density of total protein in control lane from SDS-PAGE gel

Si = Density of protein in sample lane from immunoblot

and

Ci = Density of protein in control lane from immunoblot

### Data analysis

Restricted maximum likelihood analyses (REML) were conducted using JMP 11.0 (JMP, Raleigh, USA) to determine differences amongst heat treatments and species for each physiological plant response; photosynthetic efficiency, dead biomass and LWC. Treatment and photosynthetic pathway were fixed factors with species included as random and nested within photosynthetic pathway. Multiple comparisons were undertaken using Tukey's HSD test. The proportion of leaf senecscence was transformed using a square root function to allow the data to be normally distributed. The assumptions of normality and homogeneity of variance could not be met for other response variables despite trying a range of transformation options, however, the REML method is robust with regard to non-normally distributed data. To test if there were differences in the protein extraction efficiency of our method between C4 plants and C3 plants, we analyzed the total amount of protein present in each sample (i.e. densitometry values relative to the control protein from the SDS-PAGE gels) using a REML with photosynthetic pathway as a fixed factor and species as random and nested within pathway.

Variability in individual plant responses was seen as a natural measure of the response of plant populations to extreme stress. Thus, the variances for each set of biological replicates (n = 6) for treatment and species were investigated for each of the physiological stress responses to highlight where replicate plants were responding in a highly variable manner. Variances were categorised as low, moderate and high, which were the bottom 25%, middle 50% and top 25% of variances respectively. While species were considered as random factors in the above analysis, the variation in responses by different species made it interesting to investigate species-specific responses to heat waves. The data for individual species was also found to be heterogeneous between treatments and was unable to be transformed to provide homogeneous variances. To account for the heterogeneity of our data a non-parametric analysis using the Wilcoxon signed-rank test was used for each individual species. Because analysis of the individual C3 and C4 species showed highly variable changes in the significant differences of all stress responses it was considered that the influence of the non-parametric analysis using ranking methods instead of computing variance may have extrapolated significant differences where most common parametric analyses would not [38]. As such, comparisons between individual species were interpreted with some caution.

Due to the unknown affinity of the antibodies to the different Hsp isoforms in the various grasses used in this work, direct analysis of relative Hsp expression between species was not possible. Expression of Hsp70 across stress treatments was analysed within each species using the Wilcoxon signed-rank test to account for heterogeneity of variance. Similar comparisons of Hsp17.6 amongst treatments were only undertaken in two species, *Microlaena* and *Poa*, as these were the only species where Hsp17.6 was consistently detected.

## Results

### Responses of C3 and C4 species to different stress treatments

**Fig 2:**
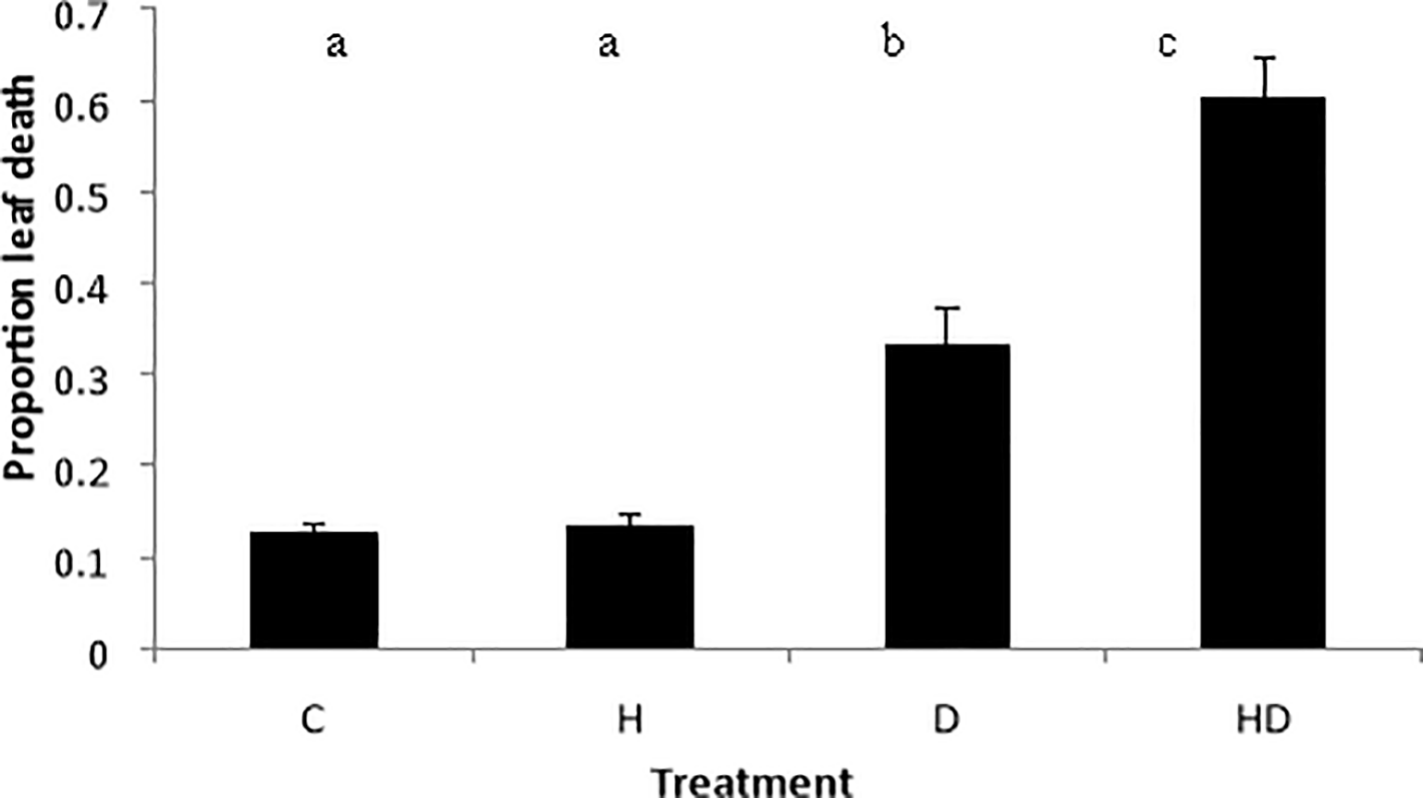
Relative leaf senescence in six native grass species after four different heat wave treatments. Leaf death was calculated as the ratio of dead dry biomass after treatment to the total dry biomass of each plant. Means are of pooled species (n = 36) with standard error. Treatments are C = control, H = heat, D = drought and HD = heat + drought. Bars not connected by the same letter denote a significant difference (p < 0.05).

C3 and C4 grasses did not differ in leaf senescence (F_1,4_ = 3.60, p = 0.127), however leaf senescence varied with heat and drought stress (F_3,12_ = 19.93, p < 0.0001; Fig 2). Overall, the HD treatment showed a significantly greater amount of leaf senescence than all other treatments losing 60% of biomass. The D treatment also showed a significant increase in leaf senescence with plants losing 31% of biomass. Greatest variance in leaf senescence was seen in the HD treatment for all species, although variation in responses for *Poa* were equivalent across all treatments (Table 1). *Austrostipa* was highly variable, relative to all other species across the three stress treatments.

**Table 1:** Variances of proportion of dead biomass to total biomass (leaf senescence), leaf water content and F_v_F_m_ for 6 species exposed to 4 different stress treatments. For each characteristic, variances are ordered by shading with the top 25% of values in dark shading, the middle 50% in light shading and the bottom 25% unshaded.

	Species	Control	Heat	Drought	Heat + Drought
Leaf Senescence					
	*Austrostipa*	0.0020	0.0310	0.0147	0.0611
C3	*Poa*	0.0032	0.0042	0.0026	0.0031
	*Microlaena*	0.0012	0.0023	0.0035	0.0201
	*Eragrostis*	0.0010	0.0081	0.0026	0.0635
C4	*Imperata*	0.0024	0.0049	0.0024	0.0863
	*Themeda*	0.0049	0.0100	0.0021	0.0142
Leaf water content					
	*Austrostipa*	0.0026	0.0015	0.0028	0.0311
C3	*Poa*	0.0003	0.0002	0.0136	0.0002
	*Microlaena*	0.0002	0.0001	0.0496	0.0337
	*Eragrostis*	0.0004	0.0003	0.0002	0.0196
C4	*Imperata*	0.0001	0.0001	0.0010	0.0515
	*Themeda*	0.0006	0.0003	0.0032	0.0087
Photosynthetic efficiency (F_v_F_m_)					
	*Austrostipa*	0.0006	0.0459	0.0002	0.0740
C3	*Poa*	0.0001	0.0073	0.0010	0.0000
	*Microlaena*	0.0001	0.0864	0.0002	0.1042
	*Eragrostis*	0.0001	0.0002	0.0001	0.0152
C4	*Imperata*	0.0004	0.0051	0.0093	0.0881
	*Themeda*	0.0003	0.0090	0.0001	0.0024

**Fig 3:**
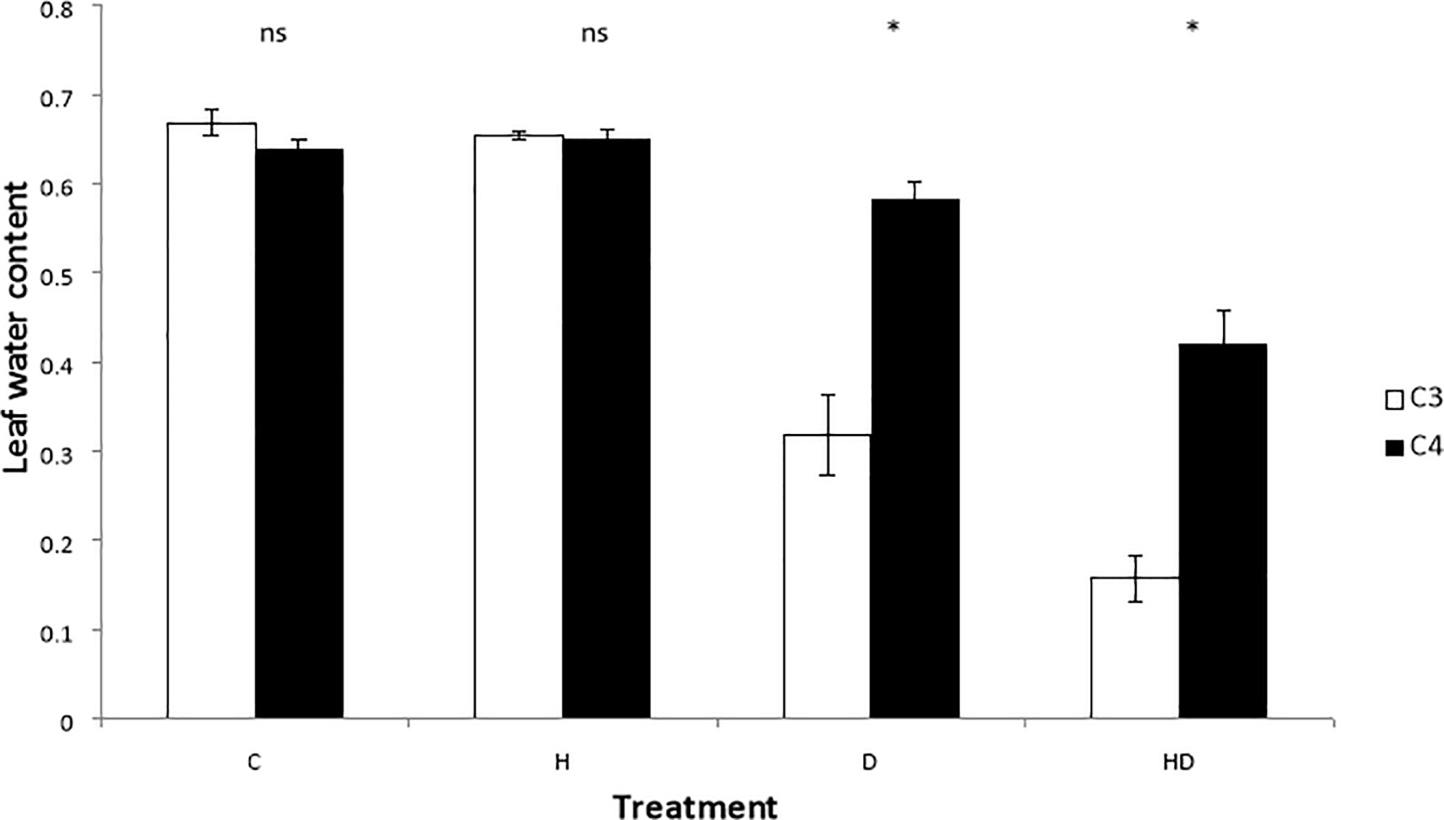
Mean leaf water content (fresh weight–dry weight, as a proportion of fresh weight) after four differing stress treatments, for Australian native grasses. Grasses are grouped by photosynthetic type (C3 or C4; n = 18+/-SE). Treatments are labelled as C = control, H = heat, D = drought and HD = heat + drought. Tukeys tests distinguished differences between means within each treatment where asterisks identify a significant difference between C3 and C4 plant types within that treatment (p < 0.05).

The effect of heat and drought stress on leaf water content varied between C3 and C4 plants (F_3,12_ = 6.103, p = 0.009, Fig 3). C3 species showed a significant decline in LWC under D treatments losing about 50% of water compared with the control. C3 plants subjected to the HD treatments had the lowest LWC at 12%. For C4 species, although declines in LWC were noted for both the D and HD treatments, no significant decrease occurred. LWC was similar between the C3 and C4 species under both the C and H treatments, however, the C3 species displayed a significant lower LWC under both D and HD treatments when compared with the C4 species. For individual species the greatest variance in LWC was seen within the HD treatment (Table 1).

**Fig 4:**
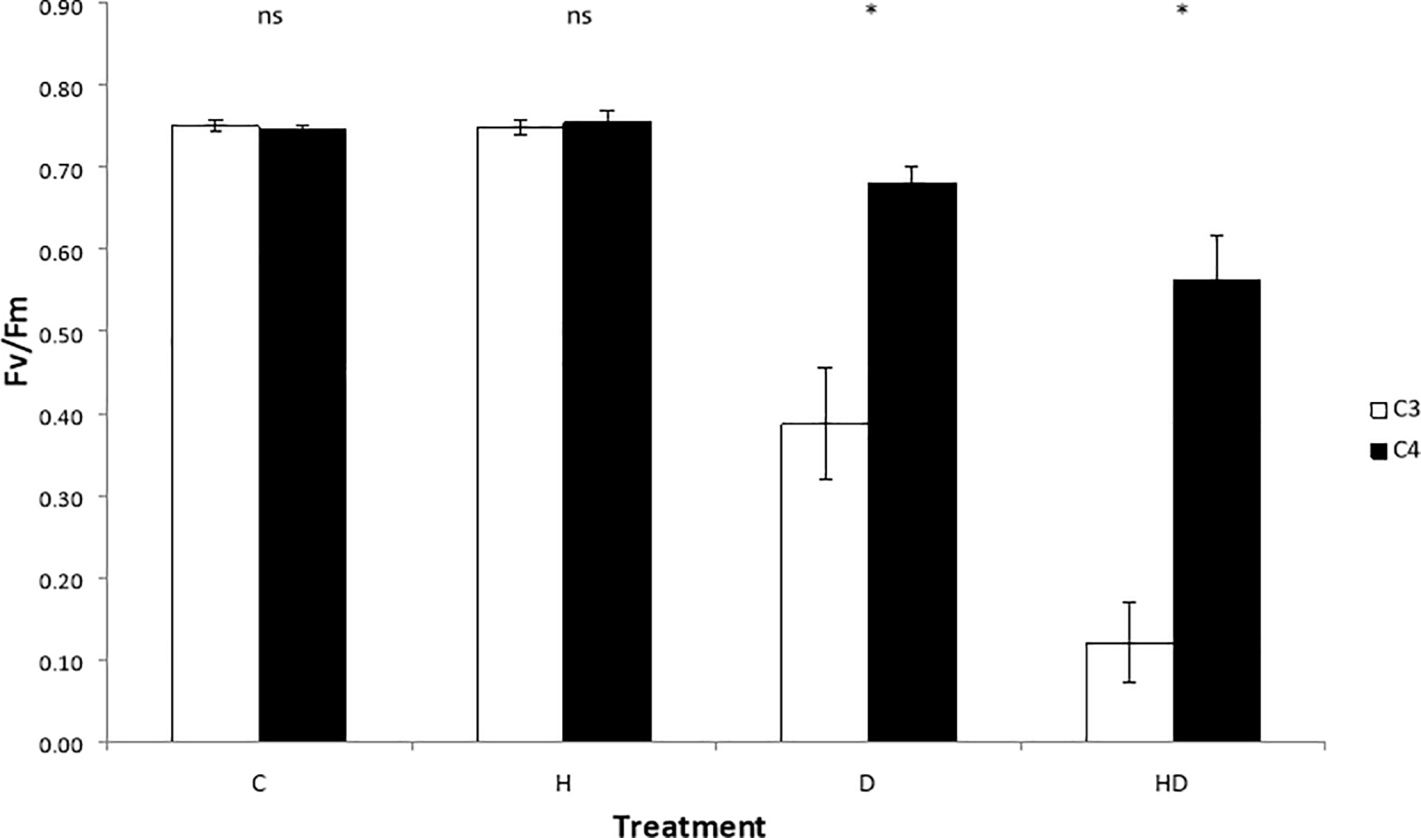
Mean maximum photosynthetic efficiency (F_v_/F_m_) after four differing stress treatments, for Australian native grasses. Grasses are grouped by photosynthetic type (C3 or C4; n = 18 +/-SE). Treatments are labelled as C = control, H = heat, D = drought and HD = heat + drought. Tukeys tests distinguished differences between means within each treatment where asterisks identify a significant difference between C3 and C4 plant types within that treatment (p < 0.05).

As expected, the effect of heat and drought stress on photosynthetic efficiency varied between the C3 and C4 groups (F_3,12_ = 5.505, p = 0.013). C3 and C4 species did not differ in photosynthetic efficiency under C or H treatments, however, C3 species displayed significantly lower photosynthetic efficiency under the D and HD treatments (Fig 4). The C4 species showed no significant change in photosynthetic efficiency in response to any of the stress treatments. For individual species the greatest variance in F_v/_F_m_ was seen within the HD treatment (Table 1).

While species were treated as random variables in these analyses, we nevertheless provide averages for each species for each treatment in the supplementary files, using Tukeys tests to identify differences amongst treatments for each species (S1, S2 and S3 Figs).

### Expression of Hsp70 and Hsp17.6 in stressed plants

**Fig 5:**
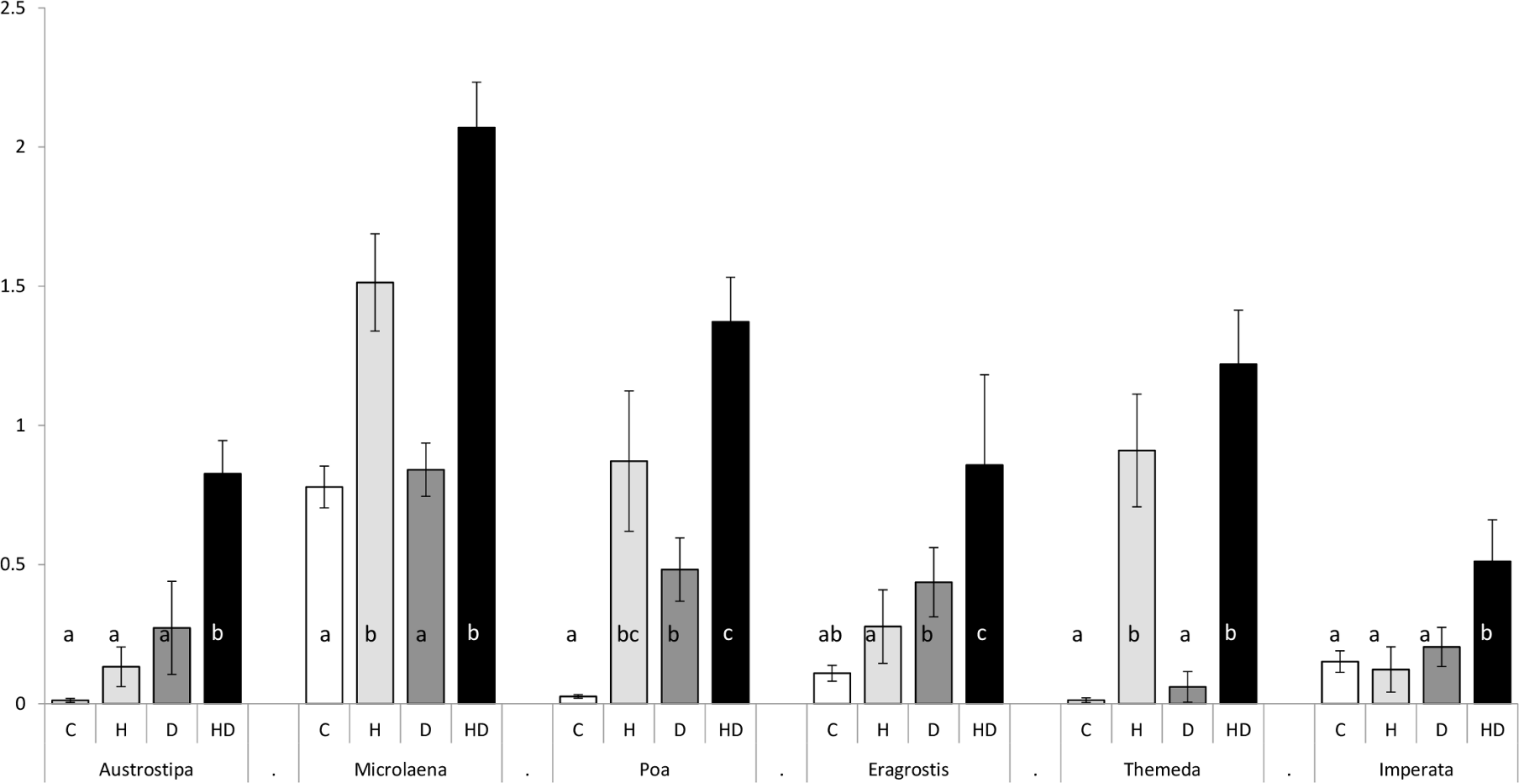
Mean relative Hsp70 expression from leaf samples of six Australian native grass species subjected to four differing stress treatments (bars made up from n = 6 biological replicates). Relative Hsp70 signal is given as a proportion of relative signal compared with the positive immunoblotting control. Grasses ordered with the top three species are C3 and the bottom three species are C4 (C3 or C4; n = 18 +/-SE). Treatments are labelled as C = control, H = heat, D = drought and HD = heat + drought. Bars within the same species not connected by the same letter denote significant difference according to the nonparametric Wilcoxon signed-rank test (p < 0.05).

The results of HSP expression were not influenced by differences in protein extraction efficiency between different photosynthetic pathways, (F_1,3.989_ = 0.073, p = 0.800). The amount of Hsp70 in leaf samples across each treatment showed a similar pattern for both C3 and C4 species (Fig 5). The amount of Hsp70 increased under both H and HD treatments in most species indicating Hsp70 responded largely to heat rather than drought stress. Compared to the control, Hsp70 expression increased significantly under HD treatment in all species except *Imperata* whereas under H alone it only increased significantly in three species (*Microlaena*, *Themeda* and *Poa*). *Poa* was the only species where Hsp70 was significantly higher in the D treatment.

**Fig 6:**
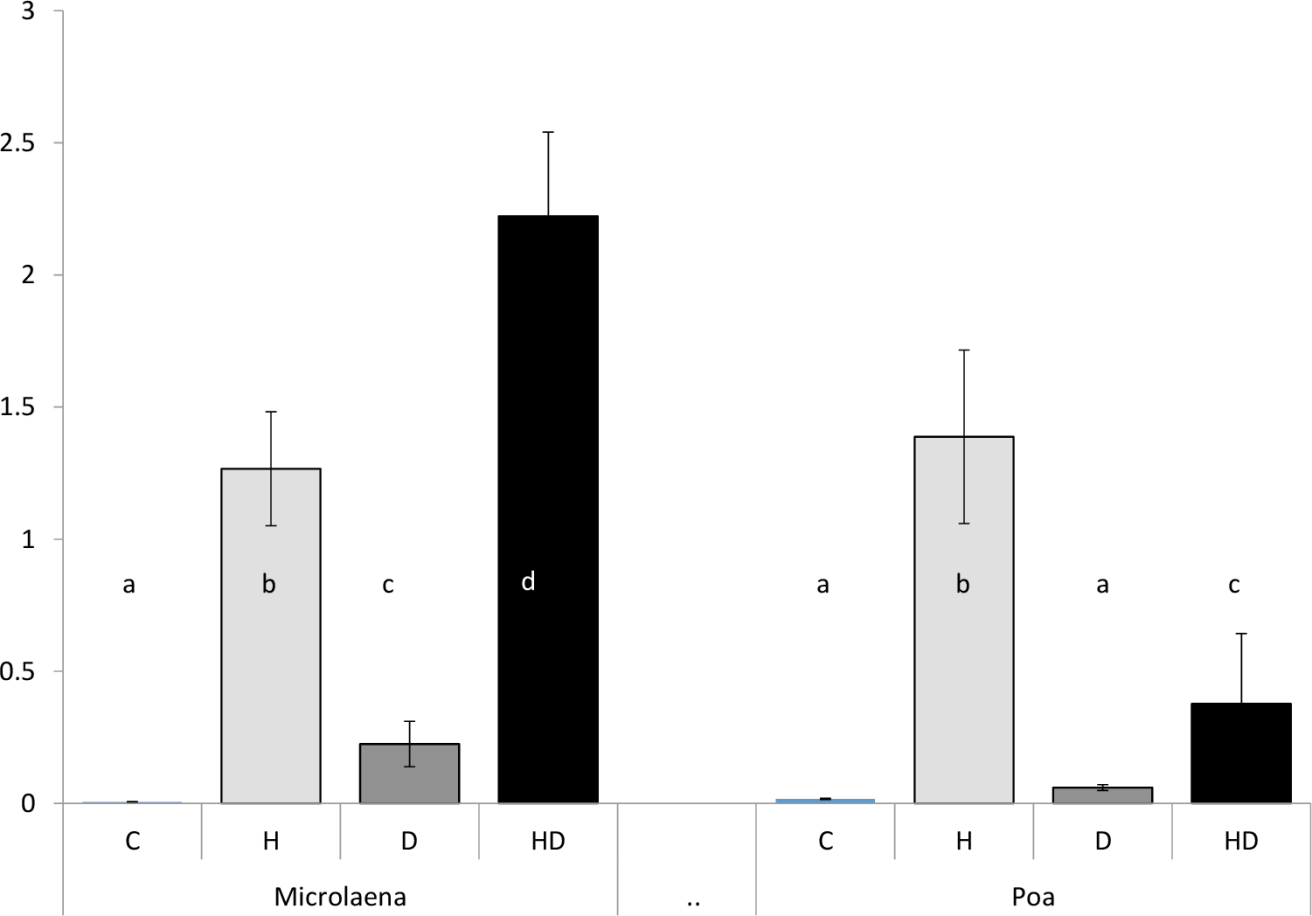
Mean relative Hsp17.6 expression, given as a value of relative signal proportional to the immunoblotting positive control. Expression is grouped for each individual species under 4 differing stress treatments for Australian native grass species *Microlaena stipoides* and *Poa labillardierei* (bars made up from n = 6 biological replicates +/-SE). Treatments are labelled as C = control, H = heat, D = drought and HD = heat + drought. Bars within the same species not connected by the same letter denote significant difference according to the nonparametri**c** Wilcoxon signed-rank test (p < 0.05).

Hsp17.6 was only consistently detected in two of the six species under experimental conditions, *Microlaena* and *Poa* both C3 species (Fig 6). In *Eragrostis* and *Imperata* Hsp17.6 was not detected within any of the treatments (data not shown). Some Hsp17.6 expression was seen in *Austrostipa* (two individuals for H and HD treatments) and in *Themeda*, combined HD treatment (data not shown).

Heat stress increased expression of Hsp17.6 in *Microlaena* and *Poa*. In *Poa*, H treatment induced a significantly greater increase in the amount of Hsp17.6 than HD treatment, although many plants in the HD treatment suffered extreme leaf damage which may have halted Hsp expression (Fig 6). In *Microlaena*, H and D treatments induced an increase in the amount of Hsp17.6 but the H treatment resulted in a much greater amount of Hsp17.6 being detected than the D treatment. Importantly, the HD treatment resulted in the highest amount of Hsp17.6 which was almost double that of H treatment alone (Fig 6).

## Discussion

### Physiological responses to heat stress

Combined heat and drought stress was far more stressful for plants than either stress alone suggesting that ecologically relevant heat waves that are usually associated with dry periods in spring and summer will be deleterious to these species. Leaf senescence showed a significantly greater increase and LWC showed a significantly greater decrease under the combined HD stress than either stressor alone. While trees are known to suffer during heat waves [39], our results suggest that native understorey grass species from warm climates will also suffer under extreme heat events associated with periods of low rainfall.

C3 plants were far less tolerant to drought stress and were particularly susceptible to the combination of affected by heat and drought. While leaf senescence did not appear to differ between photosynthetic groups, there was a significant decrease in both LWC and photosynthetic efficiency in C3 plants. When water was readily available, all species were tolerant of heat stress, however when associated with low water availability, C4 species were more tolerant than C3 species. C3 species showed significantly lower levels of LWC compared to the C4 species under combined heat and drought stress. C4 plants are able to fix more carbon with reduced water loss and are thus often better suited to hot dry environments [40–42]. This experiment provided further evidence for C4 species having a greater tolerance to some of the predicted extreme climatic shifts in the coming years [2].

It is unclear whether the leaf senescence that occurred was due to functional tolerance mechanisms or due to intolerance to the imposed stress. An argument could be made for C4 species being more tolerant to hot-dry conditions as they may simply be better able to control water loss, while the C3 species may be undergoing increased leaf senescence due to an intolerance of the leaves to high stress [43]. Given our understanding of the short-term responses developed in this work, future investigation of these plants’ ability to recover from imposed stress would contribute to understanding the functionality of this stress response and the degree to which it is being used to tolerate the stress of heat waves and droughts.

Interestingly, differences in photosynthetic pathways were not a strong predictor of response with considerable variation seen amongst species within both groups. Variation within photosynthetic groups has been identified in other studies [44, 45] and suggests that that there are factors outside of photosynthetic pathways that have a strong influence on tolerance to heat and drought stress [45]. The considerably lower tolerance observed in *Poa*, and to a lesser extent *Imperata*, under the drought and combined heat and drought stress, raises questions as to why these differences are occurring.

High variability in responses of individual plants, suggests that individuals within a species show differential heat tolerance, suggesting that some individuals will survive a heat wave while others are likely to die when the multiple stresses of a heat wave are experienced. During heat waves, high variability in leaf senescence, leaf water content and photosynthetic efficiency associated were experienced in most species. One species, *Poa*, however had low variability in these physiological measurements during heat and drought stress. *Poa* had very high levels of leaf senescence (D 81% and HD 93%) (S1 Fig). Similarly LWC (D 11% and HD 10%) and photosynthetic efficiency (D 0.06 and HD 0.007) were much lower than for all other species and clearly at the limits of survival (S2 Fig). As such individuals were all probably functionally dead, reducing the overall variability.

### Expression of Hsp70 and Hsp17.6 in stressed plants

This was the first experiment to our knowledge that compared the expression of Hsps present in leaf samples between plants of differing photosynthetic pathways. Hsp expression was generally seen to be far more responsive to heat stress than drought stress with little to no increase in Hsp70 expression seen under the drought treatments. These findings suggest that the signalling pathways for Hsp70 expression are influenced largely by heat stress and only marginally by drought stress in these native Australian grasses. In contrast, Hsp70 expression has been seen to confer tolerance for both heat stress and drought stress in a range of agricultural species [8, 21, 33] and in studies that have looked at both heat and drought stress, drought stress has played a more important role in the expression of Hsp70 [8, 33]. However, these studies have concentrated on the response to acute heat treatments and have not accounted for heat stress in the form of an ecologically relevant heat wave. Furthermore, these grass species are associated with a very dry temperate climate, where species may have evolved other mechanisms to cope with drought stress (e.g. sclerophylly where cell walls are structurally strong and unlikely to wilt). Further work is needed to elucidate if this response is seen in other native species.

The expression of Hsp17.6 was only evident in two of the C3 species (*Microlaena* and *Poa*) under the imposed stress treatments. We considered that this may be due to three possibilities: the antibodies used in the laboratory may have a higher binding affinity for the Hsp17.6 isoforms of *Microlaena* and *Poa*, the C4 species were not adequately stressed or perhaps a combination of the two. We were able to eliminate that it was due to differences in the ability to extract proteins between C3 and C4 plants by analyzing the relative amount of protein present following SDS-PAGE. Preliminary trials under acute heat stress did suggest that C4 species could express Hsp17.6, indicating that C4 species may not have been induced to express these proteins under the imposed heat regime. Barua *et al*. (2008) [46] has noted a highly diverse utilisation of Hsp expression across native Californian shrubs under heat stress. Our results add weight to the suggestion that HSP expression exhibits a highly complex and variable stress response under heat and drought stress treatments and further work is needed to identify the range of small Hsps operating in native plants.

## Conclusion

With the increasing relevance of studying the effects of climate change on plant adaptability [2], this study was unique in a number of ways. Studies on the effects of these extreme weather events have mostly been done on agricultural species with a strong emphasis on plant growth and yield. Our study is part of a growing literature which attempts to balance the emphasis of research towards better understanding the capacity of native species to tolerate the effects of increasing extreme weather events, in particular the stress imposed by heat stress, drought stress, and their combined occurrence during a heat wave. Our results have shown that if increased heat waves and drying occurs during the spring and summer months, then the detrimental effects could influence the distributions, abundances and even survival of many plant species [47, 48]. Our study investigated variation in Hsp expression within and between C3 and C4 species and was innovative in measuring the influence of heat waves with temperatures and durations that mimic natural situations, on Hsp expression of plants, rather than heat stress under shorter term laboratory regimes.

We provide additional evidence that C4 species will likely exhibit higher stress tolerance to the predicted impacts of increasing heat waves and droughts, however, much variation in stress tolerance was also observed between species of the same photosynthetic groups. More investigation of native species responses to heat and drought stress is required in order to assess species resilience under a future climate.

## Supporting information

S1 Fig Relative leaf senescence (as a proportion of total biomass) in six individual native Australian grass species (Poaceae family) after four different heat wave treatments. Means are of pooled biological replicates (n = 6) with standard error. Treatments are labelled as C = control, H = heat, D = drought and HD = heat + drought. Letters represent results from Tukeys tests undertaken on each species separately, where the same letter represents no significant difference (p < 0.05).
(DOCX)

S2 Fig Mean leaf water content (measured as in Fig 4) of six individual native Australian grass species (Poaceae family, after four different treatments. Means are of pooled biological replicates (n = 6) with standard error. Treatments are labelled as C = control, H = heat, D = drought and HD = heat + drought. Letters represent results from Tukeys tests undertaken on each species separately, where the same letter represents no significant difference (p < 0.05).
(DOCX)

S3 Fig Mean maximum photosynthetic efficiency, (F_v_/F_m_) for six individual native Australian grass species (Poaceae family) after four different treatments. Means are of pooled biological replicates (n = 6) with standard error. Treatments are labelled as C = control, H = heat, D = drought and HD = heat + drought. Letters represent results from Tukeys tests undertaken on each species separately, where the same letter represents no significant difference (p < 0.05).
(DOCX)
