# Supplementary material for: Stress in native grasses under ecologically relevant heat waves
Source: PLoS One. 2018 Oct 11;13(10):e0204906. doi: 10.1371/journal.pone.0204906 (PMC6181341; doi:10.1371/journal.pone.0204906)
Supplement: S2 Fig — Means are of pooled biological replicates (n = 6) with standard error. Treatments are labelled as C = control, H = heat, D = drought and HD = heat + drought. Letters represent results from Tukeys tests undertaken on each species separately, where the same letter represents no significant difference (p < 0.05). (DOCX) [file pone.0204906.s002.docx]

a a b a a a a a b a b b a b ac a a b

c b b a c b

S2 Fig
